# How I do it: microvascular decompression for vago-glossopharyngeal neuralgia

**DOI:** 10.1007/s00701-026-06824-4

**Published:** 2026-03-06

**Authors:** Andrei Brinzeu, Cristian Son, Marc Sindou

**Affiliations:** 1 University of Lyon, Lyon, France; 2 Timisoara Neuroscience Research Centre, Department of Neurosciences, University of Medicine and Pharmacy “Victor Babes” Timisoara, Timisoara, Romania; 3 Centre d’Etude Et Traitment de La Douleur, ELSAN, Clinique Bretéché, Nantes, France

**Keywords:** Glossopharyngeal neuralgia, Microvascular decompression, Neurovascular conflict, Vascular compression syndrome, Pain management

## Abstract

When a neurovascular conflict is demonstrated on high-resolution MRI in patients with drug-refractory Vago-Glossopharyngeal neuralgia microvascular decompression is regarded as the most effective therapeutic option. The aim of surgery is to relieve the conflict, most often produced by the posterior inferior cerebellar artery, not infrequently in association with a compressive megadolicho-vertebrobasilar artery. This can be safely achieved by exposure of the Vago-Glossopharyngeal nerve complex through a retromastoid-retrosigmoid infrafloccular approach through the cisterna magna, allowing exposure the lateral medulla and root entry zone of the lower cranial nerves. This report provides our step-by-step recommended technique for optimal efficacy and safety.

**Supplementary Information:**

The online version contains supplementary material available at 10.1007/s00701-026-06824-4.

## Introduction

Glossopharyngeal neuralgia (GN), often involving concomitant vagal rootlets (Vago-Glossopharyngeal neuralgia – VGN), is a rare disorder representing only 0.2–1.3% of facial pain, whereas trigeminal neuralgia (TN) accounts for 80–90% [10].

As primary “classical” GN arises from a neurovascular conflict, MVD offers the most durable treatment when medication fails [3, 5]. Alternative options, such as radiofrequency-lesioning or stereotactic radiosurgery, may be considered for patients unsuitable for posterior fossa surgery, but generally yield less robust long-term outcomes.

## Relevant surgical anatomy

**Fig. 1:**
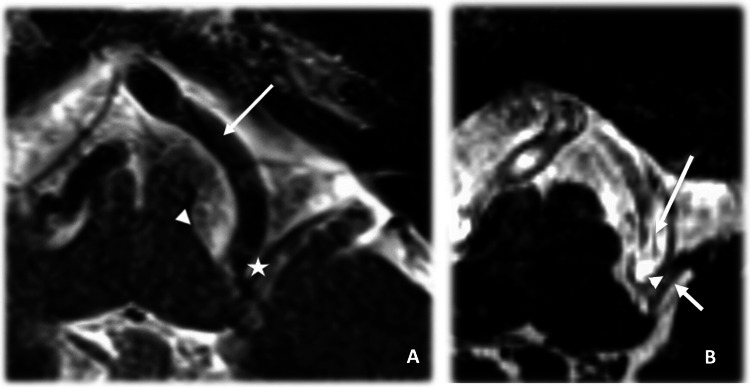
**A** Left axial T2 MR image at the medullary level demonstrating the vertebral artery (VB, large arrow) anterior to the root entry zone of the glossopharyngeal nerve (IX star), indicating a neurovascular conflict. The olivary grove is indicated by the arrow head; **B** Axial 3D CISS (T2) MR image at the level of the medulla demonstrating the vertebral artery exerting a neurovascular conflict to the glossopharyngeal nerve (IX, arrowhead). The posterior inferior cerebellar artery (PICA, small arrow) is also seen in contact with the IX nerve, suggesting an additional potential conflict

The glossopharyngeal rootlets (CN IX), which usually number one to four, emerge from the rostral portion of the post-olivary sulcus, whereas the vagus nerve (CN X) arises immediately caudal, through a longer series of 8–10 rootlets distributed along the same sulcus. Together, they converge toward the jugular foramen to enter its pars nervosa. From a surgical standpoint, this intimate arrangement explains why neurovascular compressions—from posterior inferior cerebellar artery (PICA) and/or vertebrobasilar (VB) complex—often involve both nerves simultaneously (Fig. 1).

## Description of the technique

### Preoperative planning

High-resolution MRI with T2-weighted CISS/FIESTA/DRIVE sequences is essential for surgical planning. Additional sequences, including T1 post-gadolinium (to depict veins), 3D time-of-flight (TOF) angiography (to characterize arteries), and fusion imaging, further enhance the detection of the conflict(s) [4, 9].

An otolaryngology consultation is essential to rule out local pathology that can mimic a GN. Auditory function should be evaluated to provide a baseline for postoperative comparison. Because GN may be associated with syncopal episodes, all patients undergo systematic cardiac evaluation.

### Anesthesia

Surgery is carried out under general anesthesia, usually with total intravenous anesthesia (TIVA), most commonly with propofol and remifentanil. Optimal cerebellar relaxation is achieved by limiting end-expiratory pressure and thus lowering central venous pressure. Intraoperative monitoring of the lower cranial nerves is facilitated by restricting neuromuscular blockade to the induction phase of anesthesia. Standard monitoring can be complemented by brainstem auditory evoked potentials, especially during the surgical learning curve.

### Operation room setup, installation and patient positioning

**Fig. 2:**
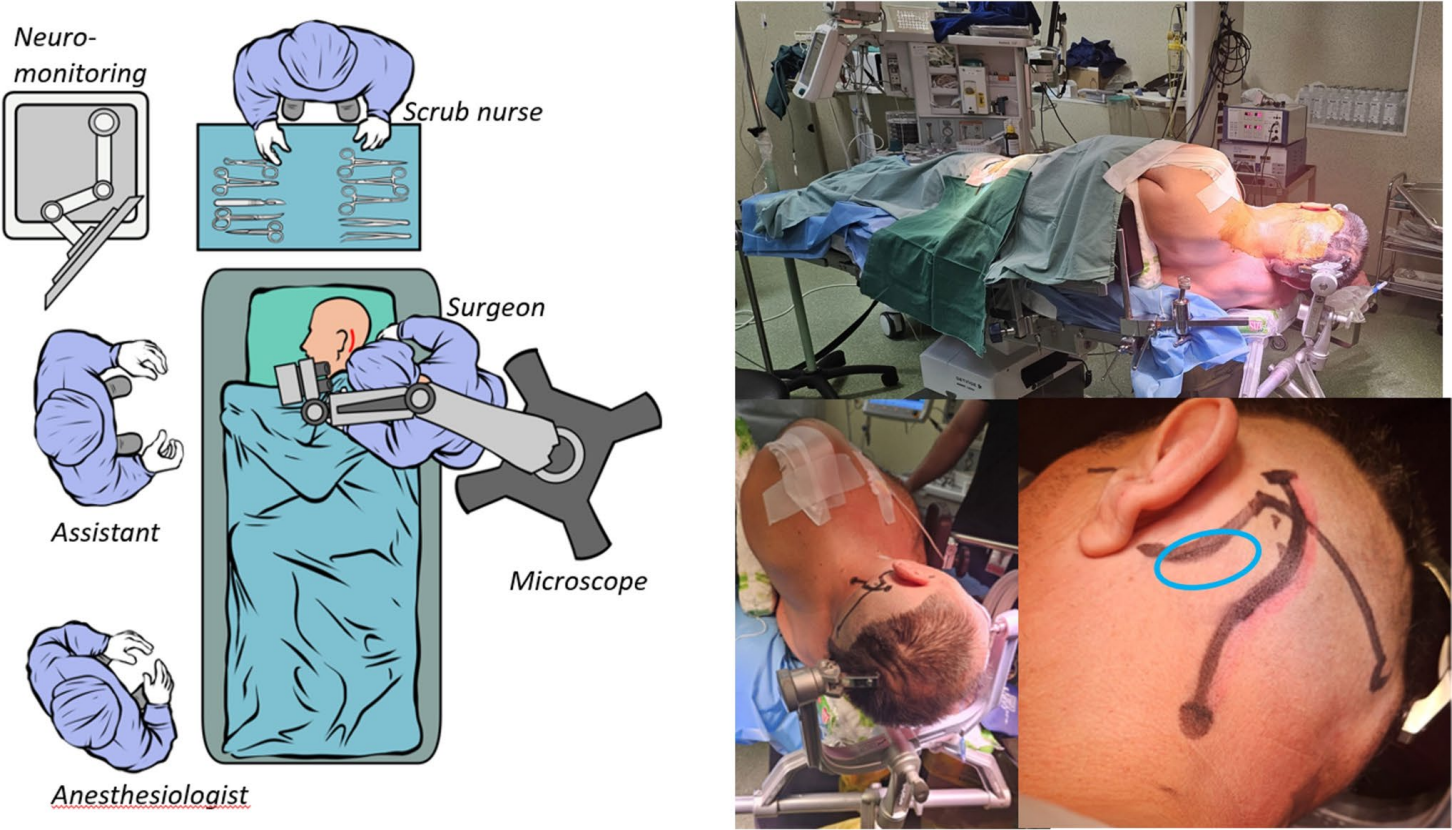
The patient is positioned in the lateral decubitus position slightly rotated backwards so that the shoulder tends to fall towards the surgeon. A back support is placed at the inferior tip of the scapulae on the midline and the torso elevated 10 degrees to facilitate venous return. The head is fixed in a three-pin head holder one pin just above the ipsilateral eyebrow and the other two on the contralateral occipital bone laterally towards the mastoid so as not to limit the surgical field and potentially provide hand support. The head is slightly flexed and, the chin kept approximately two finger-breadths from the sternum preventing venous obstruction, and rotated contralaterally. A slight lateral flexion is essential to prevent the shoulder from obstructing the surgical corridor. The shoulder is gently pulled caudally using a strap, with particular care to avoid excessive brachial plexus traction. The incision is planned posterior to the auricle, inferior to the asterional region and extended caudally toward the mastoid tip, providing optimal orientation for a caudal retrosigmoid craniectomy

We perform the procedure in the lateral decubitus position (Fig. 2).

### Microsurgical instruments

The instruments used for MVD are those typically employed for cerebellopontine angle surgery. A self-retaining retractor can be used for (gentle) cerebellar retraction; we use Sugita blades, malleable and narrow enough as to not impinge on the surgical field. [6].

Bayonet scissors allow sharp dissection of the arachnoid membranes, while fine bipolar forceps provide precise hemostasis with minimal thermal spread. A dissector probe is used to mobilize vascular loops from the IX–X rootlets and to prepare adequate space for vessel transposition or Teflon interposition, as in other cranial nerve MVD procedures.

PolyTetraFluorEthylene (PTFE)-Teflon can be either shredded or knitted and this is the currently the most used material according to the situation, Polyester Dacron is more rarely utilized [8].

### Exposure

Following the retroauricular incision, subperiosteal dissection exposes the retrosigmoid region, including the tip of the mastoid bone. If the occipital artery is encountered, it is ligated and cut. The great occipital nerve may also be encountered and is preferably mobilized since its section could lead to neuropathic pain. A keyhole retrosigmoid craniectomy is made at level of the mastoid tip. The edge of the sigmoid sinus is exposed as caudally as possible on a 3-cm length. If mastoid cells are opened, they should be occluded to prevent CSF leaks. Then, dura is opened in a curvilinear fashion, and the flap reflected to the posterior edge of the sigmoid sinus and suspended on the mastoid side.

### Approach to the vago-glossopharyngeal bundle through the arachnoid space

**Fig. 3:**
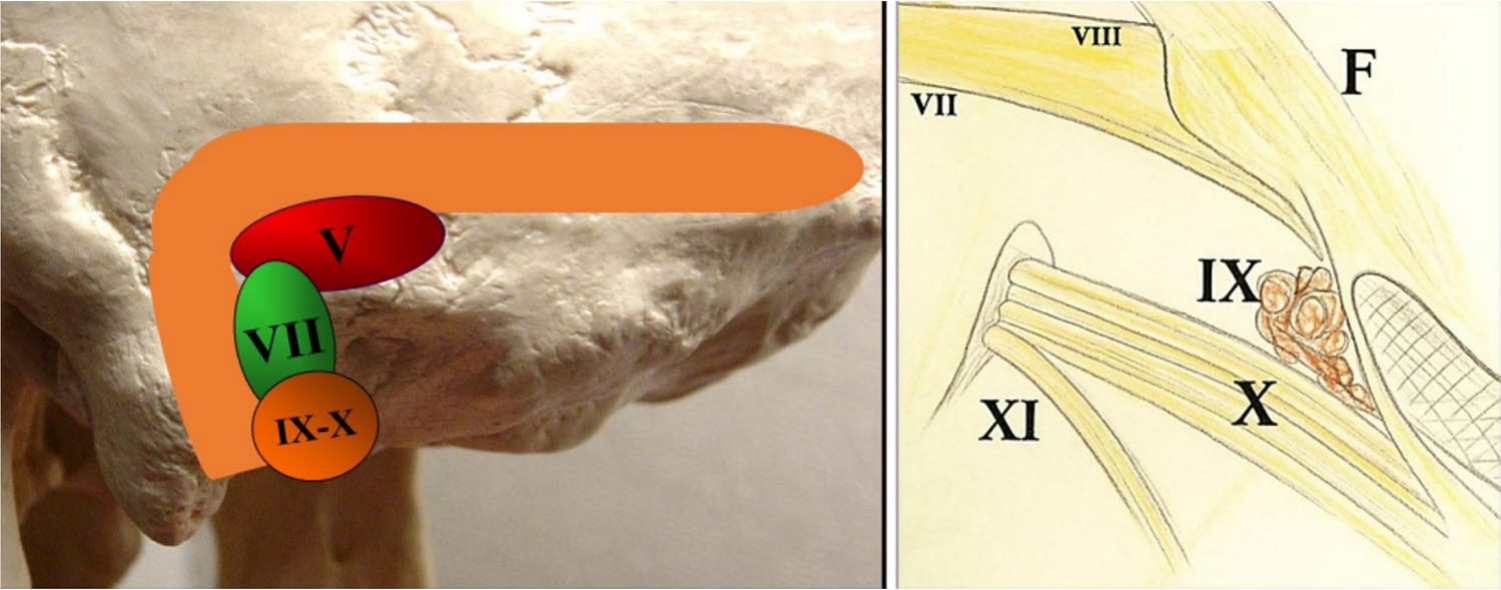
Under the operating microscope, the arachnoid membrane covering the lower cranial nerves, including the XIth (i-e the accessory nerve) is opened sharply, progressively exposing the lower cranial nerves. Dissection is carried from caudal to cranial. The glossopharyngeal and vagus nerves are to be exposed at their root entry zone in the retro-olivary sulcus and along their course toward the jugular foramen. Left: The model delineates the anatomical locations of the various craniectomies to approach the CN V, the CN VII-VIII complex, and the CN IX-X on the left side Right: The schematic drawing showing the CNs exposed through the retrocondylar craniectomy, the recommended approach for treating the neurovascular conflict of the IXth and Xth cranial nerves

After dural opening, cerebrospinal fluid release from cisterna magna allows cerebellar relaxation. A self-retaining retractor is then placed on the lateral surface of the cerebellar tonsil (exerting only minimal pressure) to create a surgical corridor toward the IX-X complex. The corridor is oriented beneath the flocculus toward the retro-olivary sulcus, allowing direct exposure of the root entry zone of cranial nerves IX and X [1] (Fig. 3).

Dissection proceeds to separating arachnoid adhesions from the neurovascular structures. Strong arachnoid adhesions are often found forming a veil covering the lower cranial nerves.

The lower cranial nerves and PICA are dissected free and exposed. When a megadolicho VBA is encountered and is compressive to the ventrolateral aspect of the brainstem, dissection starts from this artery, that must be (prudently) dislodged away first, then maintained apart using the so-called bridging technique [8].

At this stage, intraoperative neuromonitoring of the lower cranial nerves (if available) can be particularly useful to minimize the risk of postoperative dysphonia, dysphagia, or vagally-mediated cardiac disturbances [2]. Glossopharyngeal stimulation elicits pharyngeal contraction responses. Similarly, vagal stimulation produces motor activation of the vocal cords, typically monitored through endotracheal tube electrodes.

### Decompression

**Fig. 4:**
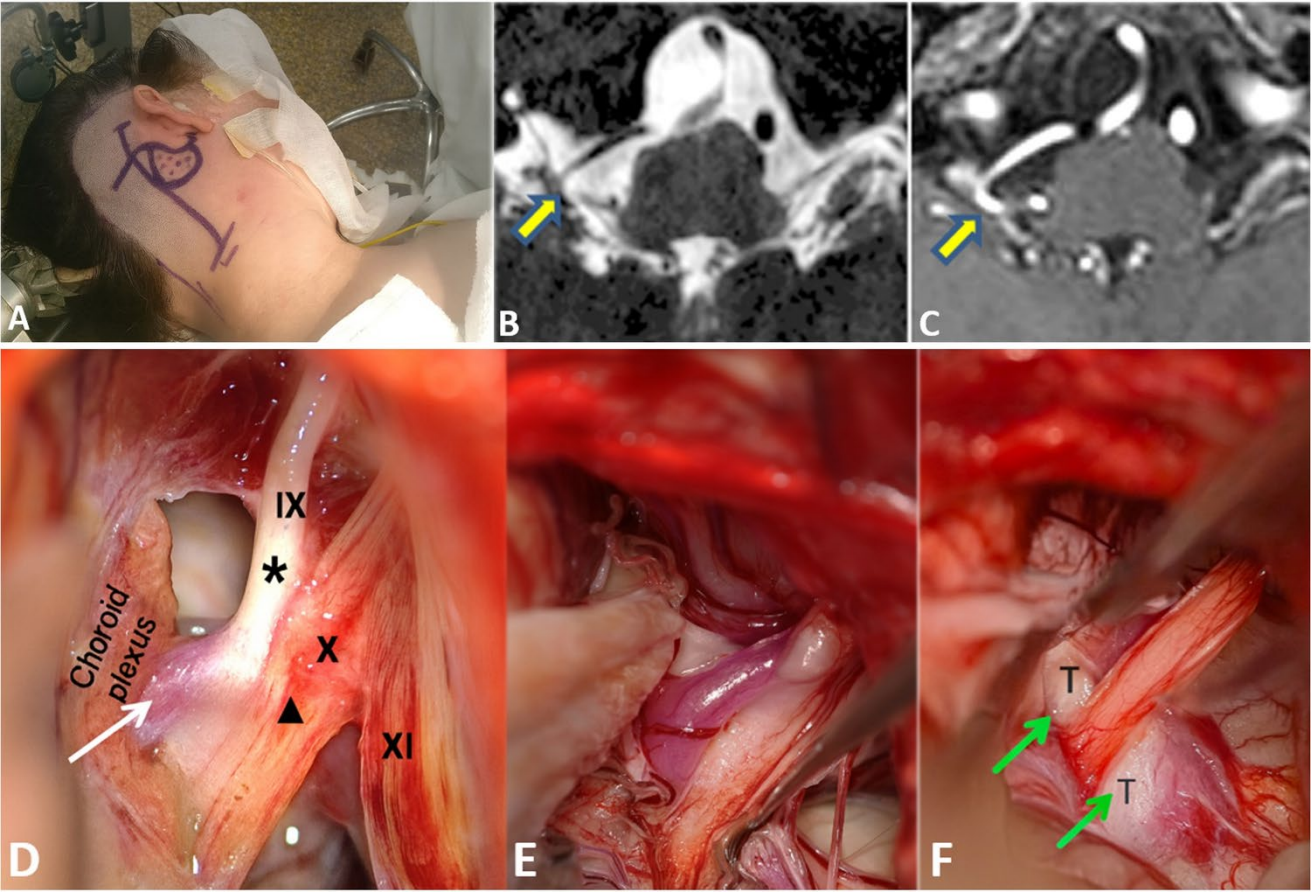
Illustrative case Patient affected with classical Vago-Glossopharyngeal neuralgia on right side. Steps of MVD surgery. **A** Landmarks of skin incision and keyhole craniectomy (retromastoid tip, retrosigmoid, infrafloccular) for approach to vagal and glossopharyngeal nerve complex for Microvascular Decompression (on right side). **B **and** C** High-resolution T2 (**B**) and + T1 gadolinium (**C**) MRI sequence. MRI shows neurovascular conflict between IXth and Xth nerve complex and posterior inferior cerebellar artery (arrow) on right side (**D**,** E **and** F**) Patient affected with a right Vago-Glossopharyngeal neuralgia due to a posterior inferior cerebellar artery (PICA) (arrow). The infra-and latero-floccular microsurgical approach (on right side) shows the offending PICA (arrowhead in D) ventral to the IXth and Xth nerves (star in D). Note the atrophic and grayish aspect of the IXth root, testifying of its focal demyelination (arrow in D). The IXth and Xth rootlets are freed from the PICA loop (**E**). PICA is maintained apart from IXth and Xth REZ (root entry/exit zone) with a piece of Teflon felt (T, arrows). The vessel is mobilized away laterally and anteriorly, allowing Teflon tapes to be interposed between the artery and the nerve to prevent direct pulsatile contact. Care is taken to avoid excessive packing that could compromise the adjacent vagus nerve. Tapes are approximatively 4 cm in length × 3 mm in width

Mobilization of the vessels can be rendered difficult by a large VBA that is fixed in place on the anterior face of the brainstem and/or by the mingling of the PICA between multiple fine rootlets of the IXth and Xth, as visible in Fig. 4.

Transposition of the offending artery(ies) is the optimal technique as – unlike interposition—it does not entail the risk of creating neocompression [7].

However, interposition of an implant material is the most used technique. Main possible side effect is the development of adhesions to the nerve that could potentially be responsible for new pain and/or recurrence.

After completion of the decompression, inspection ensures that the manipulated vessels lie free of kinking, torsion, or spasm. In the event of arterial spasm, topical application of a few droplets of papaverine diluted to 10% in saline is performed. Care is taken to avoid excessive use, given the acidic pH of papaverine (around 2.8). The combined vasodilatory effect of papaverine and irrigation with warm saline contributes to stabilization of the arteriolar microcirculation and participate to check the quality of hemostasis at the capillary level.

Venous hemostasis is verified by transient jugular vein compression and repeated Valsalva maneuvers.

### Closure

Watertight dural closure is performed by resuturing, using—if necessary—a dural patch, in our practice a small piece of autologous fascia lata and fat affixed. The osseous defect may be reconstructed with the patient’s bone powder especially in cases without mastoid cells opened. In our experience, simple closure without bone replacement has not been associated with adverse local outcomes. Finally, layered closure of the soft tissues is performed, and a compressive dressing applied.

### Postoperative care

- Care includes surveillance for potential cerebrospinal fluid leakage, rhinorrhoea through opened mastoid cells, or pseudomeningocele.
- Neurological evaluation pays particular attention to swallowing, phonation, and the gag reflex. *Transient dysphagia may happen, with patients unable to swallow solid food, relying instead on a mashed diet aided by liquids.* Symptoms rarely persist beyond three months.
- Antiepileptic medication is usually reduced by half immediately after the procedure and then progressively tapered thereafter.

## Indication

Indication for MVD is:

- Classical Vago-Glossopharyngeal neuralgia with a clear neurovascular conflict (most often PICA and/or the vertebrobasilar artery) on high-resolution MRI.
- Failure or intolerance of adequate medical therapy.
- Secondary neuralgias are in principle excluded

## How to avoid complications

- CSF release to minimize cerebellar retraction;
- Gentle arachnoid dissection to preserve CN IX, X, and XI;
- Minimal manipulation of the offending vessels;
- Decompression without compressing adjacent structures;
- Intraoperative monitoring of lower cranial nerves;
- Avoidance of excessive coagulation near the brainstem;
- Watertight dural closure to prevent CSF leakage.

## Patient information (informed consent)

- Patients are informed about the rationale for MVD, the surgical steps, and the main procedure-specific risks, including dysphagia, hoarseness, lower cranial nerve palsy, cerebrospinal fluid leak, and vascular complications.
- Alternative treatments, such as radiofrequency lesioning, stereotactic radiosurgery, or continued pharmacological management, are discussed, with an emphasis on their expected efficacy, risks, and durability relative to MVD.

## Conclusion

Microvascular decompression represents the first-line definitive surgical treatment for classical Vago-Glossopharyngeal neuralgia.

## Supplementary Information

Below is the link to the electronic supplementary material.

ESM 1 Supplementary Material 1 (AVI 334 MB)

## Data Availability

The authors declare all original data is available for review representing partial patient files as the case may apply.
